# NaMeco - Nanopore full-length 16S rRNA gene reads clustering and annotation

**DOI:** 10.1186/s12864-025-12415-x

**Published:** 2025-12-13

**Authors:** Timur Yergaliyev, Bibiana Rios-Galicia, Amélia Camarinha-Silva

**Affiliations:** 1 Institute of Animal Science, University of Hohenheim, Stuttgart, Germany; 2 HoLMiR - Hohenheim Center for Livestock Microbiome Research, University of Hohenheim, Stuttgart, Germany

**Keywords:** Nanopore, 16S rRNA gene, Metataxonomics, Clusters, GTDB, UMAP, HDBscan

## Abstract

**Background:**

Nanopore sequencing is currently one of the leading third-generation sequencing technologies on the market and is gaining popularity among researchers. Due to its long-read capabilities, full-length 16S rRNA gene metabarcoding using Oxford Nanopore Technologies (ONT) offers great potential for metataxonomic studies. However, the relatively high error rate poses a significant challenge for bioinformatic processing, often limiting taxonomy resolution to the genus level despite the longer read length.

**Results:**

This study presents NaMeco, a novel tool specifically developed to efficiently process long 16S rRNA gene reads sequenced using Oxford Nanopore Technologies, requiring minimal user input. Our tool performs read quality control, primer-specific extraction of sequences and their clustering, followed by taxonomic annotation with percent identity thresholds that minimize the amount of false-positive annotations. It produces several outputs: a table of cluster counts, taxonomic annotations of clusters, their representative sequences in fasta format and taxa counts at each taxonomy rank. Output files are compatible with the Qiime2 pipeline and can be imported into the required format for downstream analyses.

**Conclusions:**

NaMeco, in combination with the full SSU GTDB database, outperforms existing tools such as NanoCLUST and EPI2ME, while delivering taxonomy accuracy and detection rates comparable to Emu.

**Supplementary Information:**

The online version contains supplementary material available at 10.1186/s12864-025-12415-x.

## Background

Complete 16S rRNA gene sequencing with Oxford Nanopore Technologies (ONT) offers an advantage in sequence length compared to Illumina’s short-read technologies, potentially enabling more precise taxonomical annotations [1]. However, its practical application is complicated due to the relatively high error rate [2]. The longer length and higher error rate increase the number of unique reads, posing a significant challenge for downstream analyses. Moreover, clustering ONT reads to the OTUs is not only a time-consuming process but also results in either a marginal reduction in the number of unique sequences or compromises the accuracy needed for species-level annotations.

Among the tools commonly used for processing ONT 16S rRNA gene sequences, the most notable are Emu [3], NanoCLUST [4] and the EPI2ME Workflow. NanoCLUST clusters reads based on k-mer counts, selects representative sequences and, after error correction with a combination of Canu [5], Medaka and Racon [6], classifies these sequences using BLAST [7]. Unlike NanoCLUST, Emu and EPI2ME perform alignment of all the reads to reference sequences. Emu additionally employs an expectation-maximization algorithm for error correction of taxonomy assignments.

When selecting a tool, users prioritize stability, the completeness of databases, and the output generated by the selected tool. EPI2ME utilizes the 16S rRNA gene database from the National Center for Biotechnology Information (NCBI) [8]. Similar to EPI2ME, NanoCLUST uses NCBI as its default; however, the actual version of the tool is currently outdated and does not support the latest versions of NCBI. This limitation requires either the use of older databases or the modification of the tool’s configuration file. Additionally, NanoCLUST assigns species-level annotations to sequences even when the percent identity values are low. This approach leads to “top-hit” taxa that may not accurately reflect the true taxonomy of the query sequences. Emu utilizes its own database (v3.4.5, accessed 15.01.2025), which consists of sequences from the NCBI RefSeq 16S rRNA gene database and rrnDB [9]. However, some taxonomic updates are missing in this version, as subspecies recently promoted to the species level, such as *Bacillus spizizenii* and *Fusobacterium animalis*, are still classified as *Bacillus subtilis* and *Fusobacterium nucleatum* subspecies. Unlike EPI2ME, both NanoCLUST and Emu, by default, report only relative abundances at the taxonomy levels. For Emu, absolute abundances can be estimated as a fraction of total classified reads, and for the NanoCLUST, recalculated by summarizing cluster counts based on their taxonomy classification. Despite their capabilities, Emu, NanoCLUST and EPI2ME only report abundances at the taxonomy level and do not support the calculation of phylogenetic alpha and beta diversity metrics, limiting their utility for more comprehensive biodiversity assessments.

To address the challenges of processing long ONT 16S rRNA gene reads, we developed NaMeco, a new tool designed for simplicity and efficiency. NaMeco automatically performs quality control (QC) and extracts sequences based on primers. Then it counts k-mers and clusters sequences in “within samples” and “between samples” modes. Next, it generates representative sequences for each cluster and performs error correction (“polishing”), followed by taxonomy assignment up to the species level. The combination of “within samples” and “between samples” modes increases the resolution of diversity metrics by providing the sequences of clusters that are shared between the samples or unique within one NaMeco run. By default, our tool assigns species levels only to sequences with high percent identity values from the BLAST output, reducing the number of false-positive species annotations. Obtained clusters may be used as pseudo OTUs for alpha and beta diversity estimation, while taxonomy can be used for differential abundance tests and taxonomy barplots.

## Implementation

### Pipeline description

#### Workflow overview

**Fig. 1:**
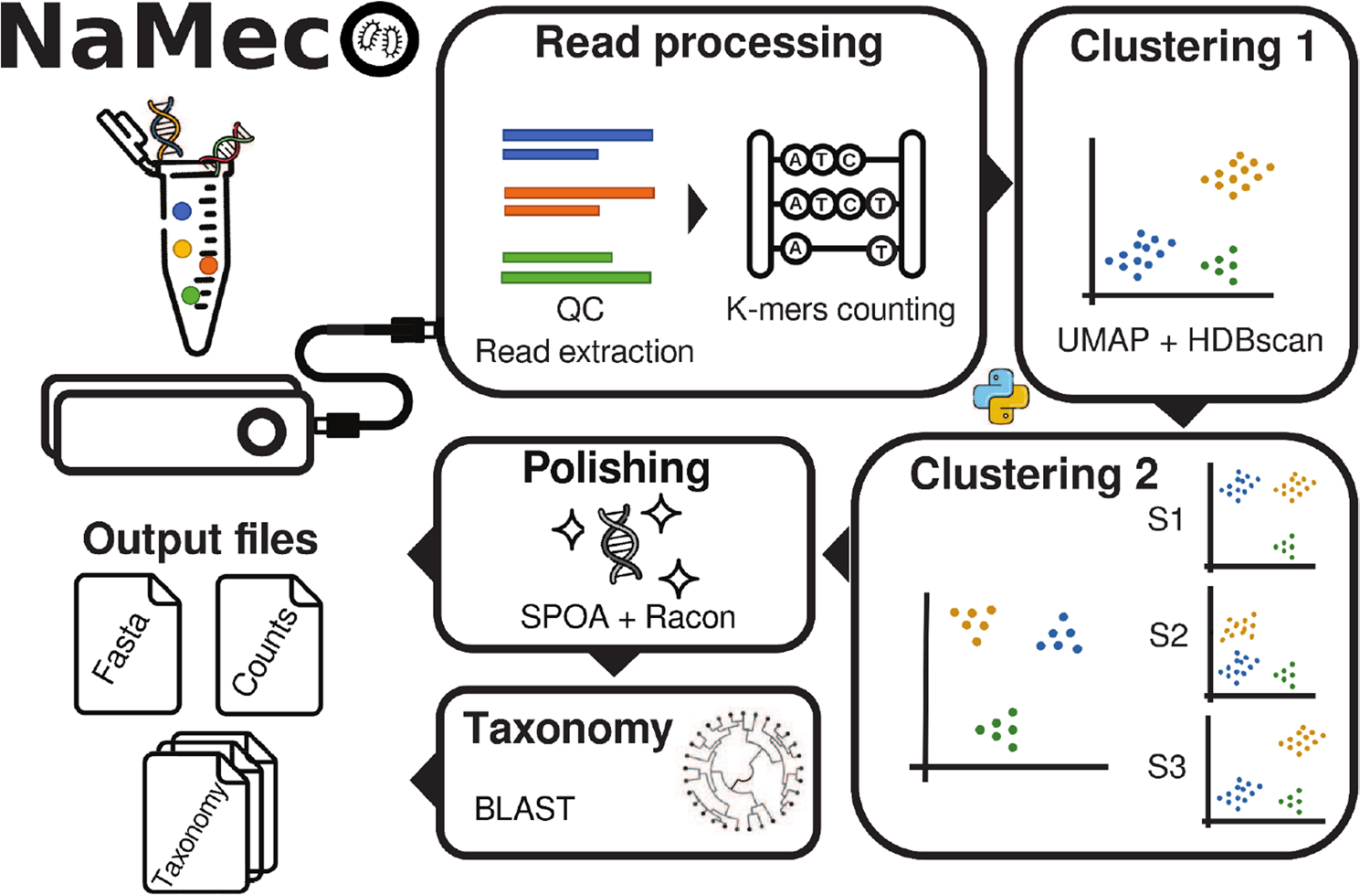
Simplified graphical representation of NaMeco workflow

NaMeco integrates various tools for read processing, polishing, and taxonomy classification (Fig. 1). It also contains “in-house” scripts for k-mers counting, recalculating counts, taxonomy selection and adjustment based on identity thresholds. It accepts a directory containing raw reads (compressed or uncompressed FASTQ files) as input. The main output files are located in the “Final output” directory: a FASTA file with representative sequences and a table with absolute counts at the cluster level, two tables with taxonomy assignments, and taxa absolute counts for each rank. Output files can be used for downstream analyses in R, Python, or other software. Representative sequences, the table with cluster counts, and one of the taxonomy tables are compatible with and can be imported into Qiime2 [10].

#### Quality control module

Quality control (QC) is an optional step and can be disabled. Otherwise, NaMeco will perform QC using Chopper [11]. Quality score and read length thresholds are adjustable. NaMeco will also extract sequences based on the primers with RESCRIPt [12] to improve read clustering. By default, it searches for the same primers that ONT uses for the amplification of the 16S rRNA gene (27 F “AGAGTTTGATCMTGGCTCAG” and 1492R “CGGTTACCTTGTTACGACTT”), but it can also accept user-defined custom primer sequences.

#### Clustering module

NaMeco counts k-mers and then clusters reads with a combination of UMAP and HDBscan [13, 14]. First, it clusters reads within each sample (“within samples” mode), then between all the samples in the run to find shared clusters (“between samples” mode). The strictness of the clustering in both modes can be adjusted by the “--select_epsilon” option, where higher values produce larger clusters but reduce sensitivity. Representative reads of each unique cluster and sample are pooled and then clustered together in the “between samples” mode. If a unique cluster consists of fewer than 100 sequences, then all sequences are used. Otherwise, 100 representative sequences are randomly selected for each unique cluster. The resulting clusters are then evaluated to find the appropriate shared cluster for each unique cluster. The shared cluster inherits all the reads from the unique cluster if at least half of the representative reads from that unique cluster are included in the shared cluster. If none of the shared clusters meet the aforementioned requirements for a given unique cluster, it is added as a shared cluster under a new cluster name. After clustering, the SIMD partial order alignment algorithm (SPOA; Smith-Waterman mode) [15, 16] creates consensus sequences for each shared cluster. To increase processing speed and decrease memory requirements, clusters with a value higher than the user-specified threshold (default 200) are randomly subsampled to that threshold.

#### Read Polishing module

In this module, NaMeco refines consensus sequences through an error correction process using Minimap2 for alignments [17] and Racon [6] for read “polishing”. These refined sequences, referred to as polished sequences, are written to the final FASTA file and serve as representative sequences. Representative sequences are used for taxonomy annotations and could also be used to calculate phylogenetic metrics in downstream analyses (between the samples within the same NaMeco run).

#### Taxonomy annotation module

This module uses BLAST to assign taxonomies to representative sequences at the species level against the SSU Genome taxonomy database (GTDB) [18]. Currently, NaMeco supports full (“All”, default) and representative species (“SpeciesReps”) versions of GTDB. Using the full version of the GTDB database increases processing time but provides more accurate taxonomy annotation. BLAST produces the output with the top 50 hits for each shared cluster. Then, all the hits are sorted in descending order based on the bit score (overall quality of an alignment) and percent identity. All hits with a bit score lower than or equal to the bit score of the best hit minus “gap” (“--gap” parameter in NaMeco settings, default 1) are discarded. To minimize false assignments, each taxonomy rank, based on the percent identity value from the BLAST output, is replaced with “unclassified” if this value is lower than a certain threshold. These thresholds were partially adapted from the previous study [19] and are 97% for species, 94.5% for genus, 86.5% for family, 82% for order, 78.5% for class, 75% for phylum and 65% for domain. Such behavior is default but can be disabled by providing the “--no_masking” option in NaMeco settings. However, we only recommend disabling this option for profiling samples with known taxonomy composition when the resulting taxonomy can be verified. The most common taxon with a fraction of all hits more than or equal to 0.6 (default value, which can be adjusted) of all retained taxonomies is selected for each cluster. Otherwise, taxonomy is selected based on the lower taxonomy rank of the taxon that satisfies selection rules, supplemented with “unclassified”. The complete taxonomies are stored in two files. In one, all taxonomy ranks are placed in the corresponding columns, one column per rank. In the second file, all ranks are combined into a single string, separated by semicolons, and prefixed with a brief abbreviation of the rank to ensure compatibility with the Qiime2 format. Additionally, cluster counts are collapsed to each taxonomy rank and stored as corresponding files in the “Final output” directory.

### Sample Preparation

#### DNA extraction and quantification

In this study, two ZymoBIOMICS™ standards were used: the Genomic DNA Microbial Community Standard (D6306; zCom; *n* = 5) and Gut Microbiome Standard (D6331; zGut; *n* = 10). DNA from the zGut standard was extracted using the Zymo DNA Mini Prep kit. DNA was quantified in a Qubit 2 Fluorometer (Thermo Fisher Scientific, USA) using QuantiFluor^®^ dsDNA System (Promega, Germany).

####  16S rRNA gene library and sequencing

Amplification of the 16S rRNA gene was conducted using the Master Mix LongAmp Hot Start Taq 2X (New England Biolabs) and the 16S Barcoding Kit 1–24 (SQK-16S024; ONT, Oxford, UK). From each sample, 10 ng of DNA was used as a target to amplify the 16S rRNA gene in a total volume of 50 µl. Amplification was performed using the following PCR conditions: one cycle of initial denaturation at 95 °C for 1 min, 25 cycles of 95 °C for 20 s, 55 °C for 30 s, and 65 °C for 2 min, followed by a final extension at 65 °C for 5 min.

Amplified barcoded DNA was quantified using a Qubit fluorometer and pooled in equimolar ratios. Pooled samples were purified using AMPure^®^ XP beads (Beckman Coulter) and quantified with a Qubit fluorometer. A total of 50 fmol of DNA was incubated with 1 µl of Rapid Adapter solution at room temperature for 5 min to prepare the DNA library (final volume: 12 µl). The library was prepared according to the protocol for Priming and loading the MinION Flow Cell R10 version (FLO-MIN114; ONT) with 37.5 µl of Sequencing Buffer, 25.5 µl of Loading Beads, and 12 µl of the DNA sample. Sequencing was performed on a MinION™ Mk1B device and MinKNOW Software version 24.06.8 (ONT) was used for base-calling and data acquisition.

### Running pipelines

All the tools tested were used with their default settings unless specified otherwise. NanoCLUST and EPI2ME were applied with the 16S rRNA NCBI database (accessed 04.10.2024). NaMeco was used in combination with GTDB database (v220) and disabled the “masking” of taxonomy annotations. Emu was tested with two databases: its default EmuDB (v3.4.5) and GTDB.

### Accuracy metrics calculation and statistical analyses

For performance evaluation, accuracy metrics, such as observed to expected taxa ratio (OET), taxonomy accuracy rate (TAR), taxonomy detection rate (TDR), Bray-Curtis distances to the standard (BC) and regression p-values and r^2^-values of observed and expected relative abundances were calculated within the Qiime2 environment using q2-evaluate-composition plugin [20]. TAR and TDR were defined as the fraction of observed taxa that were expected and the fraction of expected taxa that are observed, respectively.

Comparisons of accuracy metrics were performed using the ANOVA test [21], followed by a Tukey HSD test [22]. Letters highlighting the absence or presence of statistical difference (compact letter display) were assigned and plotted with the Python3 package cld4py (https://github.com/timyerg/cld4py*).*

## Results

### Assessing ZymoBIOMICS™ standards taxonomies

To evaluate the performance of different tools, we utilized ZymoBIOMICS standards, specifically the Genomic DNA Microbial Community Standard (zCom) and the Gut Microbiome Standard (zGut). To ensure the accuracy of the taxonomic identification, we updated the taxonomy of the isolates present in both standards by retrieving isolate names and corresponding NCBI taxonomy. Taxonomy mismatches were detected in two isolates (Table 1). Isolate B-354, labeled as *Bacillus subtilis* in zCom, was reclassified as *Bacillus spizizenii* [23]. Isolate, B-1840, originally *Lactobacillus fermentum* and present in both standards, was renamed to *Limosilactobacillus fermentum* [24].

We also used BLAST to classify 16S rRNA gene sequences [25, 26] from both standards against the GTDB. All sequences were annotated with percent identity values ranging from 99 to 100%, with identical taxonomies for all copies from each isolate. Excluding the two previously mentioned isolates, mismatches between GTDB annotations and species names declared in the standards were detected for two more isolates (Table 1). Isolate 2/1/50A, listed as *Fusobacterium nucleatum* in zGut, was classified by GTDB as *Fusobacterium animalis*. Originally, *F. animalis* was a subspecies of *F. nucleatum*, however, genome analyses led to its reclassification as *F. animalis* [27]. BLAST searches against NCBI, Emu database (v3.4.5) and Silva 138.2 [28] confirmed this classification, with all top hits corresponding to “*Fusobacterium nucleatum* subsp. *animalis”*. Isolate, OB21 TSA 19, labeled as *Clostridium perfringens*, was reported in the GTDB as *Sarcina perfringens*.

Based on the updated taxonomic information collected and described in Table 1, B. *subtilis* and *Lactobacillus fermentum* are further referred to as *B. spizizenii* and *Limosilactobacillus fermentum*. Species *F. nucleatum* was referred to as F. animalis* with an asterisk, since the taxonomy of the isolate is still not updated on the NCBI, and *C. perfringens* kept its original name, as used in the standard (Table 1).

**Table 1:** Zymo standard NCBI taxonomy and BLAST results

Species	Standard	Isolate / strain	NCBI isolate taxonomy	GTDB All SSU (BLAST)	Name in the manuscript
*Lactobacillus fermentum*	zCom, zGut	B-1840	*Limosilactobacillus fermentum*	*Limosilactobacillus fermentum*	*Limosilactobacillus fermentum*
*Bacillus subtilis*	zCom	B-354	*Bacillus spizizenii*	*Bacillus spizizenii*	*Bacillus spizizenii*
*Fusobacterium nucleatum*	zGut	2/1/50A	*Fusobacterium nucleatum*	*Fusobacterium animalis*	Fusobacterium animalis*
*Clostridium perfringens*	zGut	OB21 TSA 19	*Clostridium perfringens*	*Sarcina perfringens*	*Clostridium perfringens*

### Accuracy evaluation with zCom

DNA from the zCom was sequenced in five replicates. For taxonomy barplots and accuracy metrics calculation, only taxa with a relative abundance of ≥ 1% and detected in at least two samples were considered. In the EPI2ME output, reads annotated as “Unknown” (≅ 30%) were removed. In Emu’s output, *B. subtilis* was replaced by *B. spizizenii*, as Emu’s database has not yet updated the taxonomy.

**Fig. 2:**
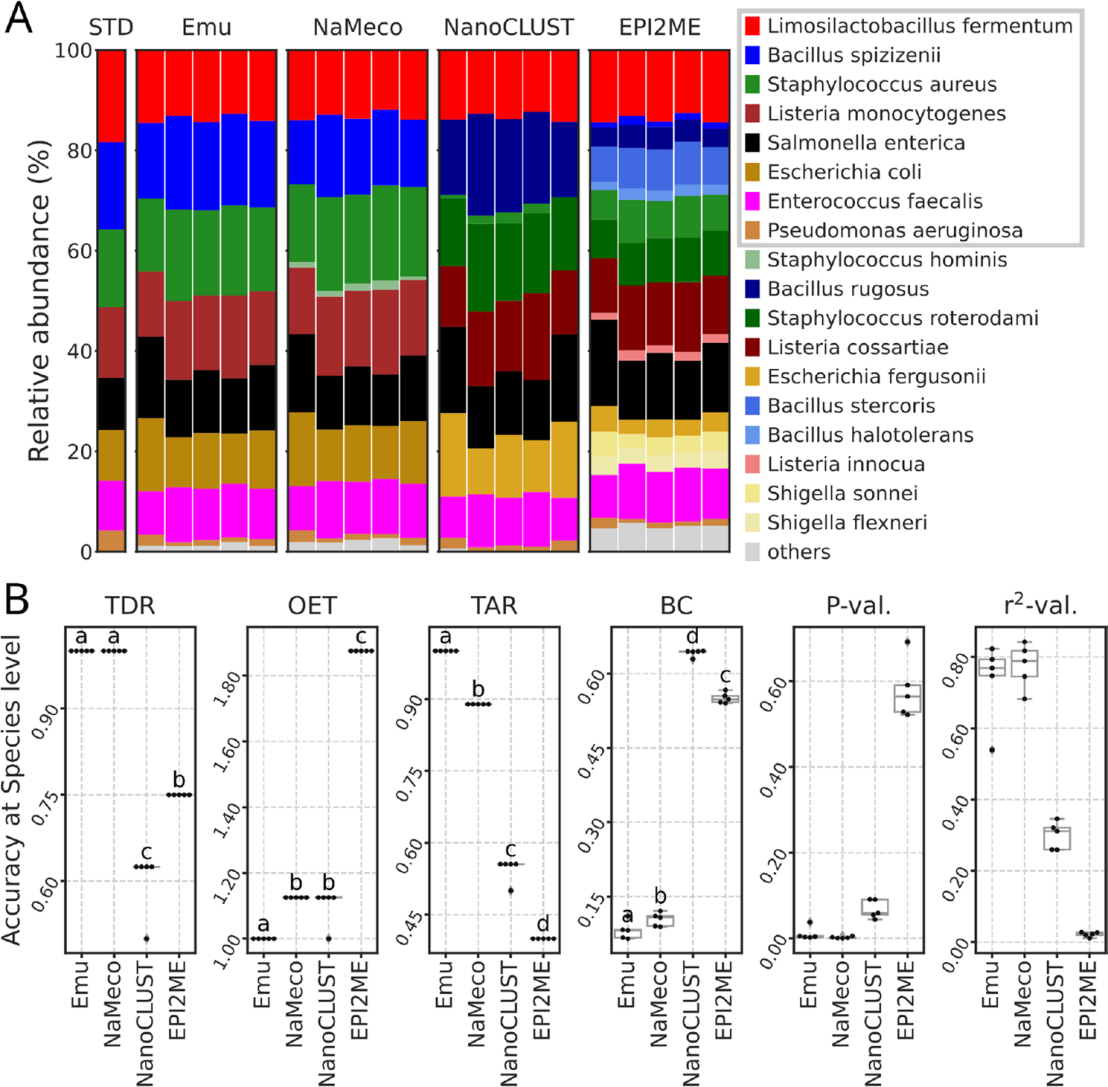
Taxonomy classification and its accuracy at the species level based on the zCom standard (D6306). **A** Barplots based on taxonomy annotations and theoretical profile from the standard. The grey rectangle in the legend highlights the taxa present in the standard. Taxa with a relative abundance lower than 1% are shown as “others”. **B** Comparison of accuracy metrics (OET - observed/expected taxa, TAR - taxonomy accuracy rate, TDR - taxonomy detection rate, BC - Bray-Curtis distances, *P*-val and *r*^*2*^-val *P* and *r*-squared values from relative abundances regression test). Only species with relative abundance ≥ 1% were considered. Letters indicate significant differences based on the adjusted *p*-values from the Tukey HSD test (*P*-adj < 0.05 for groups with different letters and *P*-adj ≥ 0.05 for groups with shared letters) and ordered by the mean (for OET and BC in ascending order and for TAR and TDR in descending)

Both NaMeco and Emu accurately identified all species (Fig. 2A). EPI2ME failed to detect two species: *Listeria monocytogenes* and *Escherichia coli* (Supplemental Table 1), and NanoCLUST three: *L. monocytogenes*, *E. coli* and *B. spizizenii*. Both NaMeco and Emu achieved an optimal taxonomy detection rate (TDR) of 1 for all samples (P-adj > 0.05). In comparison, EPI2ME and NanoCLUST exhibited lower TDR values, with EPI2ME scoring 0.75 (all P-adj < 0.001) and NanoCLUST 0.6 +/- 0.056 (all P-adj < 0.001) (Fig. 2B). 

False positive detections varied across tools. Emu did not report any false positives, while NaMeco reported one extra species - *Staphylococcus hominis*, with an average relative abundance (av. rel. ab.) of 1.23%. NanoCLUST incorrectly reported four species with av. rel. ab. ranging from 12.8 to 17.4%. EPI2ME exhibited the highest number of false positives, misidentifying nine species with av. rel ab. from 1.7 to 12.4%. A complete list of incorrectly identified taxa is available in Supplemental Table 2. These discrepancies were reflected in the observed-to-expected taxa ratio (OET). Emu had the lowest OET of 1 (optimal) across all samples compared to other tools (all P-adj < 0.001). NaMeco followed with an OET of 1.125 for all samples, while NanoCLUST had an OET of 1.1 +/- 0.056 (P-adj NaMeco-NanoCLUST = 0.176). EPI2ME demonstrated the highest OET, reporting nearly twice the number of species expected in the standard (1.875 for all samples, all P-adj < 0.001).

The taxonomy accuracy rate (TAR) varied significantly among tools (all P-adj < 0.001) (Fig. 2B). Emu achieved the highest accuracy at 1.0, followed by NaMeco at 0.889, NanoCLUST at 0.544 +/- 0.025, and EPI2ME at 0.4. Bray-Curtis distances (BC) to the theoretical profile revealed that Emu most accurately reconstructed bacterial composition (0.081 +/- 0.018, all P-adj < 0.05), followed by NaMeco (0.104 +/- 0.014, all P-adj < 0.001), EPI2ME (0.55 +/- 0.011, all P-adj < 0.001) and NanoCLUST (0.642 +/- 0.007, all P-adj < 0.001). Regression analysis of relative abundances confirmed that NaMeco and Emu produced the most accurate taxonomic profiles, with r^2^ values close to or higher than 0.7 (for all samples P-values < 0.05).

### Accuracy evaluation with zGut

Extracted DNA from the zGut was sequenced in ten replicates. Taxonomy barplots were generated by including only taxa with a relative abundance of at least 0.1%. Since zGut includes low-abundance taxa, no filtering based on relative abundances was applied for accuracy metrics calculations. Taxa detected in only one sample were excluded. Sequences classified as “Unknown” (≅ 30%) were removed from EPI2ME output. According to Table 1, F. *nucleatum* species was renamed to F. animalis*.

**Fig. 3:**
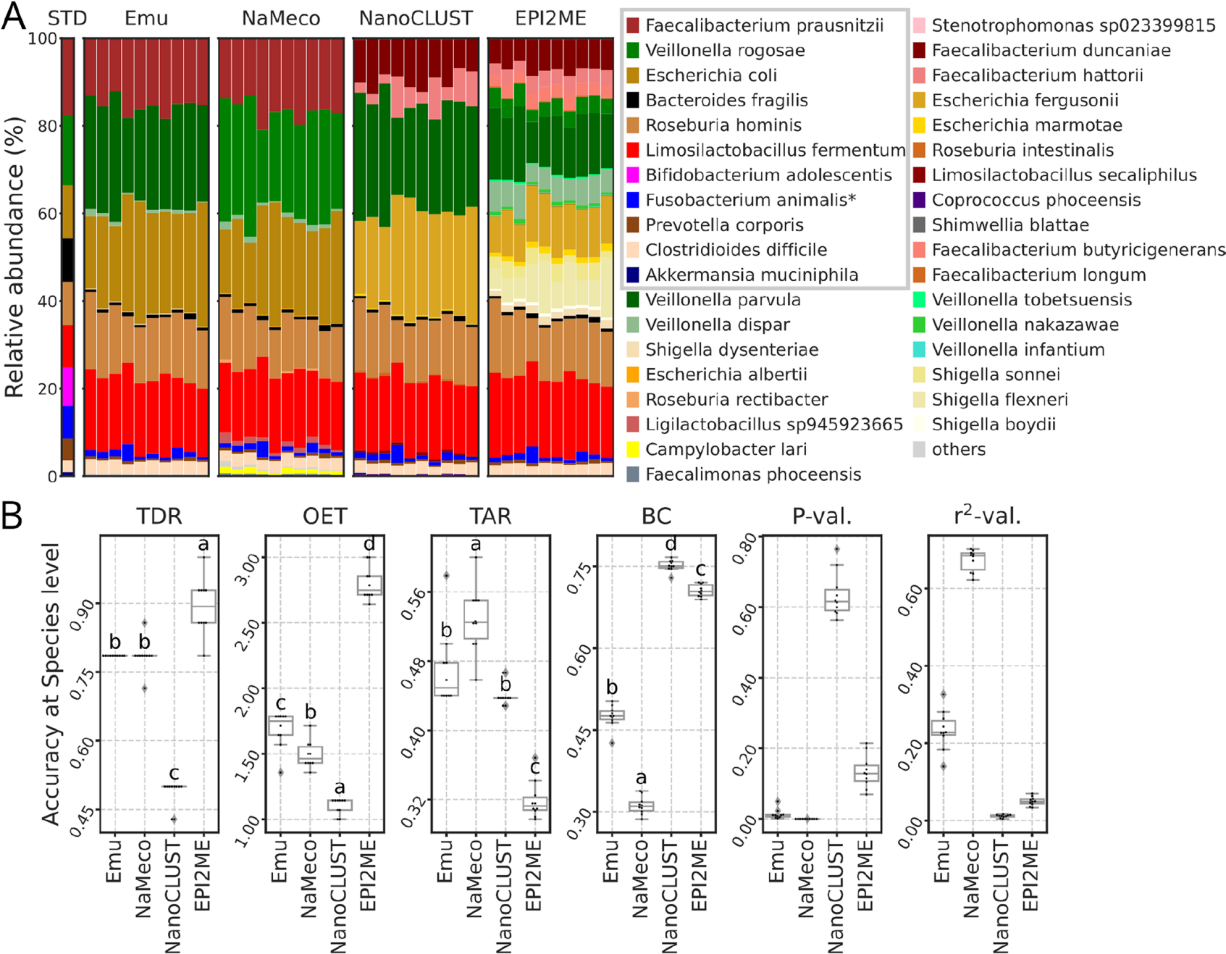
Taxonomy classification and its accuracy at the species level based on the zGut standard (D6331). **A** Barplots based on taxonomy annotations and theoretical profile from the standard. The grey rectangle in the legend highlights the taxa present in the standard. Taxa with a relative abundance lower than 0.1% are shown as “others”. **B** Comparison of accuracy metrics (OET - observed/expected taxa, TAR - taxonomy accuracy rate, TDR - taxonomy detection rate, BC - Bray-Curtis distances, *P*-val and *r*^*2*^-val *P* and *r*-squared values from relative abundances regression test). Letters indicate significant differences based on the adjusted p-values from the Tukey HSD test (*P*-adj < 0.05 for groups with different letters and *P*-adj ≥ 0.05 for groups with shared letters) and ordered by the mean (for OET and BC in ascending order and for TAR and TDR in descending)

EPI2ME was the only tool that detected all the species, including those in very low abundance (Fig. 3A-B). NaMeco failed to detect *B. adolescentis* and *C. perfringens*. Emu was unable to report three species and NanoCLUST seven (Supplemental Table 1). As a result, TDR was the highest for EPI2ME (0.893 +/- 0.061, all P-adj < 0.001), followed by NaMeco (0.786 +/- 0.034) and Emu (0.786, P-adj NaMeco-Emu = 1). NanoCLUST had the lowest TDR values (0.486 +/- 0.03, all P-adj < 0.001).

Albeit demonstrating the highest TDR, EPI2ME also reported the largest numbers of taxa not present in the zGut standard, with 34 misidentified species (Supplemental Table 2), with av. rel. ab. up to 13.4%. Emu misclassified 14 species, mostly with very low relative abundances (up to 0.6%) and one with 23.3%. NaMeco incorrectly identified 13 species (av. rel. ab. up to 1.16%) and NanoCLUST 9 (av. rel. ab. up to 24.6%). Based on it, all the tools demonstrated significantly different OET values (all P-adj < 0.005). NanoCLUST yielded the lowest OET (1.1 +/- 0.06), followed by NaMeco (1.493 +/- 0.104), Emu (1.686 +/- 0.14) and EPI2ME (2.8 +/- 0.125).

In terms of TAR, NaMeco achieved the highest taxonomy accuracy rate (0.528 +/- 0.038, all P-adj < 0.005), followed by Emu (0.469 +/- 0.044) and NanoCLUST (0.442 +/- 0.014, P-adj Emu-NanoCLUST = 0.224). EPI2ME had the lowest TAR values (0.319 +/- 0.022, all P-adj < 0.001). The Bray-Curtis distances to the theoretical zGut standard demonstrated that NaMeco was the most precise in reconstructing species composition (0.311 +/- 0.016, all P-adj < 0.001). Emu had larger BC distances (0.475 +/- 0.021) compared to NaMeco (P-adj < 0.001) but performed better than EPI2ME (0.706 +/- 0.011, P-adj < 0.001) and NanoCLUST (0.751 +/- 0.01, P-adj < 0.001). Regression analysis of relative abundances outputted by NaMeco and those from the standard produced significant P-values for all samples (all *P* < 0.001). Emu yielded significant P-values for 9 out of 10 samples (*P* < 0.05). NanoCLUST and EPI2ME generated only non-significant P-values. All r-squared values were above 0.6 for NaMeco but remained below 0.35 for the other tools.

### The impact of the database on taxonomy accuracy

The accuracy of taxonomic classification is highly dependent on the choice of reference database. Emu’s default database (EmuDB v3.4.5) is outdated and sequences of some recently assigned *Bacillus* species, such as *B. spizizenii*, are still listed as *B. subtilis*. Similarly, sequences belonging to *F. animalis* sequences as *F. nucleatum* subsp. *animalis* remain labeled as *F. nucleatum*. To evaluate the impact of database selection on performance, we tested Emu with the full SSU GTDB (v220) database and compared its accuracy to that of NaMeco. The same datasets were used (5 replicates of zCom and 10 replicates of zGut) following identical filtering criteria as in previous analyses.

**Fig. 4:**
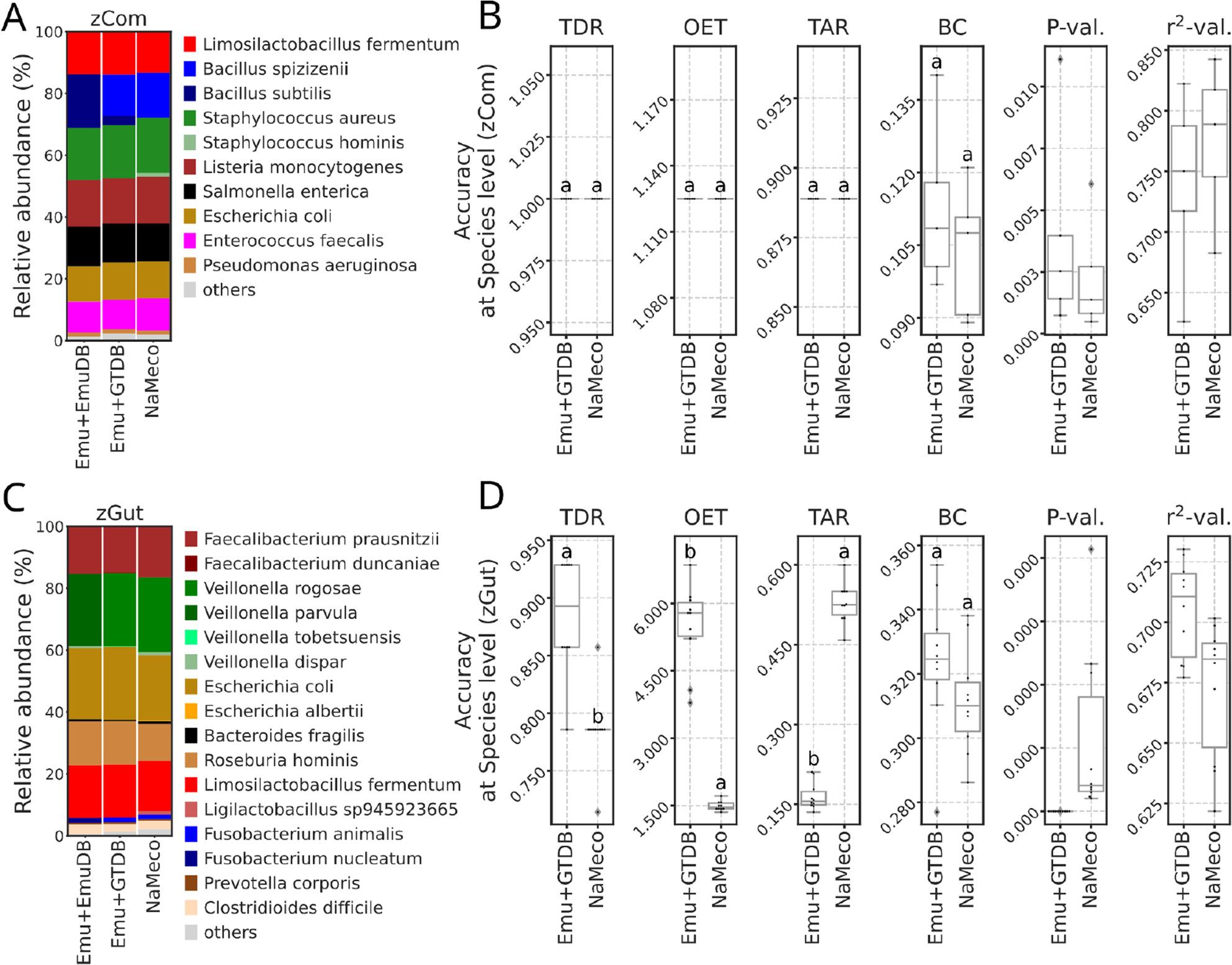
Comparison of Emu’s taxonomy classifications with standard database (v3.4.5) and GTDB (v220) and NaMeco at the species level. **A**, **C** Barplots based on taxonomy annotations from the zCom and zGut standards. Emu+EmuDB contains only original taxonomy names without renaming. Taxa with a relative abundance lower than 1% for zCom and 0.1% for zGut are shown as “others”. **B**, **D** Comparison of accuracy metrics (OET - observed/expected taxa, TAR - taxonomy accuracy rate, TDR - taxonomy detection rate, BC - Bray-Curtis distances, *P*-val and *r*^*2*^-val *P* and *r*-squared values from relative abundances regression test). Letters indicate significant differences based on the p-values (*P* < 0.05 for groups with different letters and *Pj* ≥ 0.05 for groups with shared letters) and ordered by the mean (for OET and BC in ascending order and for TAR and TDR in descending)

In prior analyses, species names in standards and Emu outputs were adjusted to reflect updated nomenclature (*B. subtilis* to *B. spizizenii* and *F. nucleatum* as F. animalis*) to perform fair comparisons. However, in this section, taxonomy barplots (Fig. 4A, C) for Emu with its default database (Emu + EmuDB) retain their original taxonomy assignments. For the zCom standard, Emu, using EmuDB continued to classify *B. spizizenii* reads as *B. subtilis* (Fig. 4A). When using the GTDB database, Emu classified most of the reads of that isolate as *B. spizizenii*, providing an updated name. However, some of the reads were still annotated as *B. subtilis*. NaMeco accurately annotated *B. spizizenii* but also reported *S. hominis* as an additional species. Despite these minor differences, no significant variations in accuracy metrics were detected between Emu + GTDB and NaMeco (Fig. 4B).

For the zGut standard, Emu with EmuDB misclassified *Veillonella rogosae* as *Veillonella parvula*, while Emu with GTDB correctly classified it (Fig. 4C). A similar discrepancy was observed for *Fusobacterium species*. *F. nucleatum* was classified as *F. nucleatum* with the original database and *F. animalis* with GTDB. This reassignment aligns with Table 1, where BLAST searches using GTDB classified all 16S rRNA gene copies of *F. nucleatum* isolates from the standard as *F. animalis*. A separate BLAST search against the Emu database revealed taxonomy entries labeled as *F. nucleatum subsp. animalis*, reinforcing the inconsistency caused by outdated taxonomy records.

Emu with GTDB had a higher TDR (0.886 +/- 0.05) compared to NaMeco (0.786 +/- 0.034, *P* < 0.001) (Fig. 4D). However, this improved detection rate came at the expense of specificity, with Emu with GTDB yielding a much higher OET (5.5 +/- 0.939) compared to NaMeco (1.493 +/- 0.104, *P* < 0.001). This suggests that Emu + GTDB may overpredict species, leading to an inflated number of detected taxa. In contrast, NaMeco produced higher TAR values of 0.528 +/- 0.038, significantly outperforming Emu + GTDB, which had a TAR of 0.165 +/- 0.026 (*P* < 0.001). Bray-Curtis distances to the theoretical standard did not significantly differ between Emu + GTDB and NaMeco. Regression analysis of relative abundances confirmed that both tools produced statistically significant P-values and high r-squared values.

### Primer-specific read extraction

**Fig. 5:**
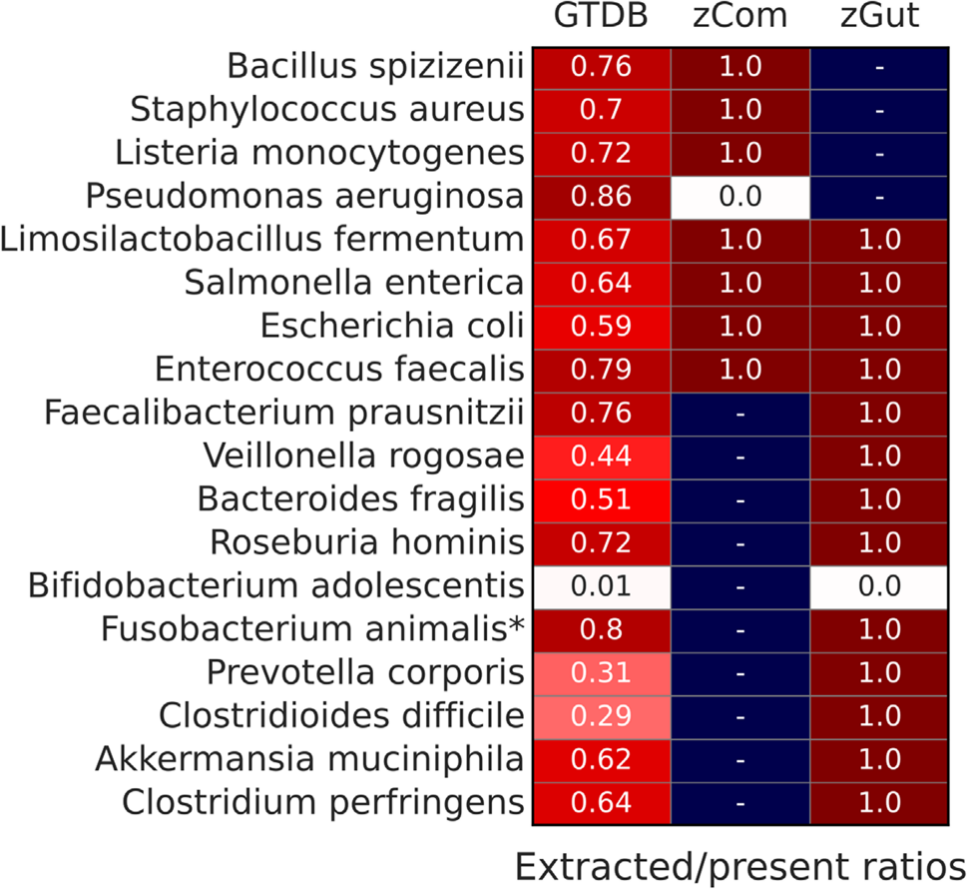
Extracted/present ratios of zCom and zGut species. These ratios were calculated for each species as a fraction of all 16S rRNA gene copies of standard species that were extracted from the GTDB database or standard with ONT primers

To investigate whether ONT primers efficiently amplify 16S rRNA gene sequences from specific bacterial species, we analyzed primer-specific extraction performance using a 95% identity threshold. Several bacteria, including *Pseudomonas aeruginosa* from zCom standard, and *B. adolescentis*, *Bacteroides fragilis*, F. animalis* and *Prevotella corporis* from zGut were consistently reported in lower than expected abundances. We extracted reads matching the ONT 16S rRNA gene primer sequences from two datasets: (i) all species present in both standards from GTDB database and (ii) all 16S rRNA gene copies from the zCom and zGut standards (Fig. 5). We then calculated the ratios of extracted reads relative to the total number of available sequences for each species.

The results indicate that none of the 16S rRNA gene copies from *P. aeruginosa* and *B. adolescentis* were extracted from their respective standards. However, when applied to the GTDB database, 0.86 of *P. aeruginosa* sequences were successfully extracted, suggesting that the standard’s specific *P. aeruginosa* isolate may contain primer mismatches. In contrast, only 1% of *B. adolescentis* sequences from GTDB were retrieved, indicating a more pronounced primer bias against this species.

## Discussion

### ZymoBIOMICS™ standards

The high sequence conservation of the 16S rRNA gene in some bacterial species often limits the resolution needed to differentiate closely related taxa. For example, species like *B. spizizenii*, *B. stercoris* and *Bacillus inaquosorum*, previously *B. subtilis* subspecies, were reclassified to the species level [23]. However, many bacterial species still contain clades or subspecies that show minimal or inconsistent variation in the 16S rRNA gene sequences. The above-mentioned factors highlight the importance of tool and database selection for taxonomic annotations. For example, isolate B-354 (NCBI references CP118021 and CP118022) (Table 1), listed as *B. subtilis* in the zCom standard, is currently classified as *B. spizizenii* [23]. While Emu with default database (v3.4.5) continued to annotate this isolate as *B. subtilis*, its classification was corrected to *B. spizizenii* when using GTDB (v220) (Fig. 4A). NanoCLUST and partially EPI2ME, which rely on NCBI database, misclassified the same isolate as *B. rugosus*, likely due to the high sequence similarity between these taxa (>99.93%) [29]. Similar classification issues arise with other species in the standard. *E. coli* and *L. monocytogenes* were misannotated by NanoCLUST and EPI2ME as *E. fergusonii* and *Listeria cossartiae*, respectively. Given that *E. coli* and *E. fergusonii* have approximately 64% DNA-DNA hybridization [30], and *L. monocytogenes* and *L. cossartiae* share over 99.9% sequence similarity in their 16S rRNA gene [31], these misclassifications show the difficulty of distinguishing closely related taxa using 16S rRNA sequencing, independently of the tool or algorithm applied.

A similar pattern was observed in the zGut standard. NanoCLUST and EPI2ME misannotated *Faecalibacterium prausnitzii* as *Faecalibacterium duncaniae* and *Faecalibacterium hattorii* (Fig. 4C), which were recently assigned to the species level, and diverge from *F. prausnitzii* [32]. Additionally, *Veillonella rogosae* was incorrectly classified as *Veillonella parvula* by these tools and by Emu when using its default database, while NaMeco and Emu with GTDB correctly identified it. Difficulties in correctly identifying *Veillonella* species based on the 16S rRNA gene are a well-documented issue [33, 34].

The considerations described above led to the selection of the GTDB as a database for NaMeco. Additionally, when considering alternative 16S rRNA gene databases, such as 16S-ITGDB [35], Silva [28], EzBiocloud [36], RNAcentral [37], MIMt [38], RiboGroove [39] and RDP [40], the GTDB database stood out by offering a strong combination of key features: thorough species level taxonomic curation, up-to-date taxonomy nomenclature, a high number of representative sequences, and long-lasting support with regular updates.

To mitigate classification errors, NaMeco incorporates several user-adjustable parameters that improve annotation confidence, such as the “--gap” parameter, minimum fraction for top-hit selection and masking taxonomies based on percent identity thresholds (see Pipeline Description). If no single taxon meets the specified fraction (“--min_fraction”, default 0.6 for all ranks) for top-hit taxonomic selection, the output is adjusted to a lower taxonomy level with the prefix “unclassified”.

These features enhance NaMeco’s reliability in species-level annotations, contrasting with tools like NanoCLUST, which lack similar parameters against annotation inaccuracies. Nevertheless, caution is recommended when interpreting species-level annotations with any tool and database based solely on 16S rRNA gene sequences. The results demonstrate that NaMeco, paired with GTDB database, outperforms tools that rely on NCBI database.

### Taxonomic accuracy

By default, NanoCLUST applies a minimum cluster size threshold of 100 reads, which reduces noise but limits its sensitivity. Consequently, NanoCLUST reported the lowest OET values for zGut, which includes rare taxa, but not for zCom, where all species are abundant (Figs. 2B and 3B). However, it should be noted that authors recommend adjusting this parameter according to the desired level of sensitivity [4]. In contrast, EPI2ME and Emu classified all individual sequences, leading to a higher number of rare taxa. In the zCom dataset, where rare taxa were absent, we applied a relative abundance threshold of 1% for accuracy metrics calculation, and Emu yielded a perfect OET score of 1, while EPI2ME reported the highest OET due to misclassification (Fig. 2B). The Emu’s EM-based algorithm for error correction in alignments likely contributed to its accuracy [3]. However, when analyzing data from the zGut standard, which includes rare taxa, no relative abundance filtering was applied. As a result, both Emu and EPI2ME produced the highest OET values (Fig. 3B). Testing Emu with the GTDB further increased OET values (Fig. 4D), reporting five times more species than expected. To balance sensitivity with specificity, we set NaMeco’s default minimum cluster size to ten reads, ensuring reliable detection without excessive false positives.

NaMeco and Emu had the highest TDR for zCom, correctly identifying all the species, while EPI2ME and NanoCLUST underperformed due to misclassified species (Fig. 2A-B). For zGut, EPI2ME had the highest TDR values, correctly classifying all the species in at least part of the samples. Although *E. coli* species were detected by all tools, its abundance was underestimated by EPI2ME (~ 0.24% vs. 12% in the standard), with most reads misclassified as *E. fergusonii*. In contrast, NaMeco and Emu reported *E. coli* on an average of 21.1 and 22.9%, respectively. Higher than expected relative abundances in these tools may be due to primer biases and DNA extraction inconsistencies, discussed in the next section. *B. adolescentis*, expected to constitute ~ 8.8% of the zGut standard, was entirely undetected by NaMeco, Emu and NanoCLUST and was found in only two out of ten samples by EPI2ME with an extremely low relative abundance of less than 0.001%. This suggests that EPI2ME’s higher TDR does not reflect true sensitivity, as it also reported nearly three times more taxa than expected (OET ≈ 2.8).

For zCom, Emu with its default database had higher TAR than NaMeco (Fig. 2B), but only after its outdated classification of *B. spizizenii* as *B. subtilis* was corrected. (Table 1). For zGut, NaMeco outperformed Emu in TAR (Figs. 3B and 4D), with fewer false positives and a correct classification of *V. rogosae*, which was misidentified as *V. parvula* by Emu with its default database.

Regression analysis of observed vs. expected taxon relative abundances confirmed that NaMeco and Emu (with both databases) yielded the highest r-squared values and lowest p-values, indicating the strongest agreement with the theoretical standard compositions. Moreover, both tools successfully reconstructed the species composition of the standards, exhibiting shorter Bray-Curtis distances to standards.

### DNA extraction and primer specificity

Previous research reported that ONT primers (27 F “AGAGTTTGATCMTGGCTCAG” − 1492R “CTACGGTTACCTTGTTACGACT”) perform suboptimally for some species, including *Bifidobacterium spp.* [41, 42]. Our primer-specific sequence extraction failed to retrieve *B. adolescentis* sequences from zGut (Fig. 5), and succeeded only for 1% of *B. adolescentis* sequences from GTDB. Obviously, the lack of *B. adolescentis* in the tools reports is related to the primers used in the ONT 16S rRNA gene barcoding kit, which do not correctly amplify its 16S rRNA gene sequences [1].

Another species whose sequences were not extracted by the ONT primers from the standard was *P. aeruginosa*. However, 86% of *P. aeruginosa* sequences were retrieved from the GTDB by the same primers. This species is present in the zCOM with a relative abundance of ~ 4%, but was reported by all the tools with a relative abundance of 1.5-2%. A similar trend is shown in Supplemental Fig. 4 of the NanoCLUST publication [4]. Since zCom is a DNA standard that eliminates DNA extraction bias. We hypothesize that the 16S rRNA gene copies of *P. aeruginosa* isolate B-3509, used in the zCom standard, are not efficiently targeted by ONT primers.

We initially expected that the most underrepresented species in our dataset would belong to groups known to be challenging to lyse. However, *Bacteroides fragilis*, F. animalis*, and *Prevotella corporis* are gram-negative bacteria, which are generally easier to lyse than gram-positive species [43, 44]. Therefore, their low relative abundance, reported by all tools tested, could be attributed to biases introduced during DNA extraction or PCR amplification with 27 F-1492R primers.

## Conclusions

NaMeco demonstrated good overall performance with ZymoBIOMICS standards. It outperformed NanoCLUST and EPI2ME and showed comparable or superior accuracy to Emu, depending on the reference database and evaluation metric.

This study further demonstrates the advantages of using GTDB database over NCBI for species-level taxonomy assignments of 16S rRNA gene sequences obtained from ONT MinION sequencing.

By offering representative sequences, taxonomic annotations, and cluster-based outputs, NaMeco facilitates downstream analyses such as phylogenetic diversity estimation. Additionally, its output format ensures compatibility with Qiime2, further broadening its applicability in microbiome research.

The study also identified potential biases introduced by ONT primers, which may impact taxonomic profiles. These findings reinforce the importance of primer efficiency and database selection.

Overall, NaMeco presents itself as a robust and efficient tool for microbiota analysis, offering accurate species-level profiling and enhanced taxonomic resolution for ONT long-read sequencing. 

## Supplementary Information

Supplementary Material 1.

## Data Availability

NaMeco is publicly available at: https://github.com/timyerg/NaMeco . The datasets generated and/or analyzed during the current study are available in the European Nucleotide Archive repository (ENA) under the accession number PRJEB82315.
